# Investigating the Proximity of the Lower Alveolar Canal to the Apex of Premolar and Molar Teeth in the Mandible Using Cone Beam Computed Tomography (CBCT) in Tabriz, Iran -

**DOI:** 10.31661/gmj.vi.3936

**Published:** 2025-10-20

**Authors:** Shahriar Shahi, Shirin Kolahdouz, Sahar Ghanbaran, Mohammad Gerayeli, Emad Movahed, Mohammad Ali Irani, Shaghayegh Ghadimi

**Affiliations:** ^1^ Department of Endodontics, Faculty of Dentistry,Tabriz University of Medical Sciences, Tabriz, Iran; ^2^ Dental and Periodontal Research Center,Tabriz University of Medical Sciences, Tabriz, Iran; ^3^ School of Dentistry, Shahid Sadoughi University of Medical Sciences, Yazd, Iran; ^4^ Department of Endodontics School of Dentistry, Shiraz Branch, Islamic Azad University, Shiraz, Iran; ^5^ Private Practice, Periodontist Lux Smile Dental Clinic, Mashhad, Iran; ^6^ School of Dentistry, RWTH Aachen University, Aachen, Germany; ^7^ Doctor Irani Clinic and Research Center, Tehran, Iran; ^8^ Department of Endodontics, Faculty of Dentistry,Tabriz University of Medical Sciences, Tabriz, Iran

**Keywords:** Alveolar Canal, Mandible, Molar Teeth, Premolar Teeth, Cone Beam Computed Tomography

## Abstract

**Background:**

Knowing the anatomical link between the IAN and surrounding structures is vital before endodontic operations to avoid injuring the IAN. Examining the relationship between the inferior alveolar canal (IAC) and the apices of mandibular premolars and molars using cone beam computed tomography (CBCT) is the primary objective of this work.

**Materials and Methods:**

Two hundred and twenty patients, ranging in age from sixteen to seventy-seven, who visited the University of Tabriz’s Faculty of Dentistry had their CBCT images examined in this retrospective cohort study. Mandibular fractures, pathologies, or bone syndromes were not considered, as were teeth with diseases impacting canal contact. Additionally, poorly defined IAN pictures were not included. The shortest distance between the root apex and the upper border of the interosseous capsule was determined by taking measurements using cross-sections that were 0.3 mm thick.

**Results:**

Analysis of 220 CBCT images revealed a gender distribution of 56.8% female and 43.2% male patients, with age groups of 49 years (32.3%). The greatest mean distance between the teeth and the IAC was observed in the first premolar in males (5.7 mm), while the shortest was in the third molar in females (2.91 mm). Distances from mandibular molars and premolars to the IAC showed significant differences: second and first molars had smaller distal than mesial distances (P0.05), and second premolars had greater distances on the right side (P0.001). Males exhibited greater distances than females for molars and premolars (P0.05), but age had no significant impact (P0.05).

**Conclusion:**

Mandibular premolars maintain the most significant distance, while the third molar is closest to the IAC. Gender differences are significant, while age does not impact these measurements.

## Introduction

The inferior alveolar nerve (IAN) originates in the mandible's ramus and travels laterally through the mandible's body before ending at the mental foramen in the lower jaw canal [1]. When performing endodontic treatments [2], implant insertion [3][4], or third molar extraction [5] in the mandible, it is essential to estimate the distance between the IAN and the apices of the lower jaw teeth. Paresthesia, dysesthesia, or even anesthesia and pain following treatment could be the consequence of either temporary or permanent nerve injury [6].

Root canal filling materials' neurotoxic effects, mechanical pressure from overfilling [7], and temperatures exceeding 10°C near the IAN are three of the many endodontic treatment mechanisms that can injure the IAN [8]. Some patients might experienced iatrogenic nerve damage as a result of root canal treatments [9].

Imaging modalities such as digital periapical radiography, panoramic radiography, spiral computed tomography, and CBT scans have been utilized to assess the location of the IAN and its association with demographic and anatomical factors. Recently, CBCT has been used to assess the IAN's connections to demographic and anatomical features. Research has demonstrated that CBCT consistently and accurately measures the cortical labial and lingual plates' height and thickness, making it a dependable and effective tool for pre-treatment planning linear measurements [10].

The association between the location of the IAN and various dental structures has been the subject of extensive research, which has helped to refine our understanding of this connection. Using CBCT, Vidya et al. (2019) examined 100 patients' mandibular molar positions and discovered that there were gender-based differences in linear measures between the first and second molars on both sides [11]. Using CBCT, Hiremath et al. (2016) determined that there were substantial variations in the locations of the second premolar, first and second molar apices, and the inferior alveolar canal [12]. By employing CBCT, Dabaghian et al. (2014) determined that the position of the inferior alveolar canal in relation to the roots of molars and premolars remains largely stable regardless of gender or age [13]. No matter the age or gender of an adult, the position of the inferior alveolar canal is nearly always fixed, according to Adigüzel et al. study [14].

In order to avoid damaging the IAN, which might occur during endodontic treatments, it is essential to locate the IAN in relation to any nearby structures before treatment [3][4]. There has not been a CBCT radiographic evaluation of the distance between the apices of the roots of premolar and molar teeth and the inferior alveolar canal, according to prior searches. Consequently, the purpose of this study is to use CBCT images to more precisely determine the distance between the inferior alveolar canal and the apices of the premolar and molar teeth, as well as the proximity of the IAN to the apices of the roots.

## Materials and Methods

This retrospective descriptive-analytical study was conducted on 220 CBCT images of patients aged 16 to 67 years who were referred to the Radiology Department of the Tabriz Dental Faculty. Ethical principles were fully observed, and all patient information remained confidential. Due to the inability to obtain informed consent from patients, this study was carried out after receiving approval from the ethics committee. The present study was approved by the Ethics Committee of Tabriz University of Medical Sciences under the approval number IR.TBZMED.VCR.REC.1399.552.

The inclusion criteria for the study encompassed CBCT images of the first and second premolars and the first, second, and third molars on both the right and left sides of the mandible, where the inferior alveolar canal was visible (with the presence of all premolar and molar teeth not being mandatory). The exclusion criteria included CBCT images where the inferior alveolar canal was not visible, teeth with specific pathologies affecting their relationship with the canal, and mandibular bone exhibiting fractures, pathologies, or bone syndromes.

### Sample Size Determination

The sample size was determined based on the results of Portaji et al. [15] and considering a 5% alpha level, with data entered into the sample size calculation formula to estimate the required number. To enhance the accuracy of the study, an additional 10% was added to the estimated sample size. Ultimately, 220 CBCT images were randomly selected.

### Data Collection

All measurements were performed by a dental student under the supervision of a radiology specialist using previously acquired CBCT scans. Images were obtained using a NewTom VGi device, which features a cone-shaped beam, flat-panel detector, and 360-degree rotation. The exposure time for all patients was 3.6 seconds, with a scan time of 18 seconds. The field of view was 15 × 15 cm, with a kVp of 110 and a variable current ranging from 1 to 20 mA. Measurements were conducted using the NNT Viewer software on CBCT scans in cross-sectional views with a slice interval and thickness of 0.3 mm. Images were viewed on a monitor with a resolution of 1536 × 1920 pixels, a pixel size of 127 × 127 µm², and a pixel depth of 14 bits.

### Measurement Protocol

In the cross-sectional view, the shortest distance from the root apex to the superior border of the inferior alveolar canal was measured. The inferior alveolar canal typically appears as a radiolucent circle with a diameter of up to 4 mm in cross-sectional views [16]. Cross-sectional imaging of the mandible provides three-dimensional visualization, which is more accurate than two-dimensional radiography [17]. Radiographic sections were magnified to visualize nerves and root apices clearly. To facilitate identification of the inferior alveolar canal, the orientation of cross-sectional slices was adjusted to ensure the canal was perpendicular to the coronal plane.

When the mental foramen was located beneath the premolars, the closest vertical distance from the root apex to the superior border of the mental foramen was measured. If the mental foramen was positioned posterior to the premolars, the distance from the apex of these teeth to the continuation of the IAN canal, specifically the superior border of the anterior loop or the incisive canal, was evaluated.

### Data Categorization

Participants were divided into three age groups: Group 1, under 18 years; Group 2, 18-49 years; and Group 3, over 49 years. Additionally, participants were classified by sex. Measurements from each root apex were categorized based on age and sex.

### Statistical Analysis

Results were reported as means ± standard deviations and frequencies (percentages). To compare measurements across sex and age groups, independent t-tests and one-way analysis of variance (ANOVA) were used. Data were analyzed using SPSS version 17 . The significance level was set at P<0.05.

## Results

**Table:** Table1. Gender and Age Distribution of Patients in CBCT Images

**Variable**	**Frequency (n)**	**Percentage (%)**
**Gender**		
**Female**	125	56.8
**Male**	95	43.2
**Age Group**		
**<18 years**	62	28.2
**18-49 years**	87	39.5
**>49 years**	71	32.3

N=220 CBCT images analyzed

**Table:** Table2. Mean Distances (mm) from Mandibular Molars and Premolars to the Inferior Alveolar Canal by Side

**Tooth**	**Right Side (M ± SD)**		**Left Side (M ± SD)**	
	Mesial	Distal	Mesial	Distal
**Third Molar**	2.60 ± 1.17	2.59 ± 1.03	2.35 ± 1.2	2.31 ± 0.96
**Second Molar**	3.64 ± 1.22	3.19 ± 1.43*	3.47 ± 0.99	3.04 ± 1.6*
**First Molar**	4.08 ± 1.81	3.75 ± 1.87*	3.61 ± 1.89	3.23 ± 1.72*
**Second Premolar**	4.66 ± 1.78	4.21 ± 1.54*	4.21 ± 1.54	—
**First Premolar**	5.33 ± 1.58	5.18 ± 1.25	5.18 ± 1.25	—

^*^ Significant differences between mesial and distal distances (or right and left sides for premolars) are indicated by P<0.05, based on independent t-tests or one-way ANOVA.

**Table:** Table3. Comparison of Distances from Mandibular Molars and Premolars to the Inferior Alveolar Canal by Age Group and Side

**Tooth**	**Age Group**	**Side**	**Mesial Mean ± SD (mm) **	**P VALUE (Mesial)**	**Distal Mean ± SD (mm) **	**P VALUE (Distal)**
**Third Molar**	<18	Right	2.75 ± 1.24	0.456	2.52 ± 0.97	0.521
	18-49	Right	2.59 ± 1.09		2.47 ± 1.09	
	>49	Right	2.74 ± 0.96		2.46 ± 1.26	
	<18	Left	2.51 ± 1.1	0.492	2.32 ± 0.94	0.299
	18-49	Left	2.34 ± 1.3		2.29 ± 0.98	
	>49	Left	2.56 ± 1.04		2.31 ± 1.15	
**Second Molar**	<18	Right	3.48 ± 1.17	0.258	3.16 ± 1.69	0.369
	18-49	Right	3.47 ± 1.32		3.31 ± 1.69	
	>49	Right	3.59 ± 2.34		3.41 ± 2.13	
	<18	Left	3.29 ± 0.96	0.334	2.90 ± 0.33	0.241
	18-49	Left	3.31 ± 1.48		3.29 ± 1.48	
	>49	Left	3.41 ± 1.01		3.25 ± 0.16	
**First Molar**	<18	Right	4.11 ± 2.12	0.522	4.06 ± 1.64	0.496
	18-49	Right	3.83 ± 2.05		3.52 ± 1.8	
	>49	Right	3.91 ± 1.7		3.76 ± 1.82	
	<18	Left	3.60 ± 1.57	0.226	3.39 ± 1.5	0.343
	18-49	Left	3.71 ± 2		3.57 ± 1.93	
	>49	Left	3.50 ± 1.53		3.26 ± 1.37	
**Second Premolar**	<18	Right	4.55 ± 1.53	0.524	—	0.621
	18-49	Right	4.76 ± 0.5		4.21 ± 1.19	
	>49	Right	4.65 ± 2.19		4.15 ± 1.88	
	<18	Left	4.29 ± 1.39	0.162	—	—
	18-49	Left	4.21 ± 1.19		—	—
	>49	Left	4.15 ± 1.88		—	—
**First Premolar**	<18	Right	5.41 ± 1.21	0.059	—	0.162
	18-49	Right	5.40 ± 1.27		5.10 ± 1.11	
	>49	Right	5.26 ± 1.89		5.25 ± 1.39	
	<18	Left	5.29 ± 1.76	—	—	—
	18-49	Left	5.10 ± 1.11	—	—	—
	>49	Left	5.25 ± 1.39	—	—	—

Distances are in millimeters (mm). P-values are based on one-way ANOVA comparing age groups for each measurement. Dashes (—) indicate no distal measurements for premolars, as only one measurement was provided. N=220 CBCT images analyzed.

**Table:** Table4. Comparison of Distances from Mandibular Molars and Premolars to the Inferior Alveolar Canal by Gender and Side

**Tooth**	**Gender**	**Side**	**Mesial Mean ± SD (mm) **	**P VALUE (Mesial)**	**Distal Mean ± SD (mm) **	**P VALUE (Distal)**
**Third Molar**	Male	Right	2.7 ± 1.09	0.267	2.53 ± 1.09	0.591
	Female	Right	2.69 ± 1.24		2.44 ± 0.97	
	Male	Left	2.51 ± 1.3	0.349	2.22 ± 0.98	0.622
	Female	Left	2.39 ± 1.1		2.19 ± 0.94	
**Second Molar**	Male	Right	3.68 ± 1.17	0.021	3.46 ± 1.69	0.004
	Female	Right	3.39 ± 1.26		3.11 ± 1.16	
	Male	Left	3.62 ± 0.96	0.001	3.39 ± 1.52	0.001
	Female	Left	3.11 ± 1.01		2.88 ± 1.67	
**First Molar**	Male	Right	4.2 ± 1.76	0.002	4.06 ± 1.94	0.001
	Female	Right	3.75 ± 1.85		3.64 ± 1.80	
	Male	Left	3.7 ± 1.86	0.003	3.19 ± 1.50	0.033
	Female	Left	3.31 ± 1.92		3.47 ± 1.93	
**Second Premolar**	Male	Right	4.86 ± 1.67	0.001	—	—
	Female	Right	4.45 ± 1.88		—	—
	Male	Left	4.41 ± 1.19	0.001	—	—
	Female	Left	4 ± 1.88		—	—
**First Premolar**	Male	Right	5.7 ± 1.27	0.001	—	—
	Female	Right	4.96 ± 1.89		—	—
	Male	Left	5.7 ± 1.11	0.001	—	—
	Female	Left	4.65 ± 1.39		—	—

Distances are in millimeters (mm). P-values are based on independent t-tests comparing genders for each measurement. Dashes (—) indicate no distal measurements for premolars, as only one measurement was provided. N=220 CBCT images analyzed.

**Figure-1:**
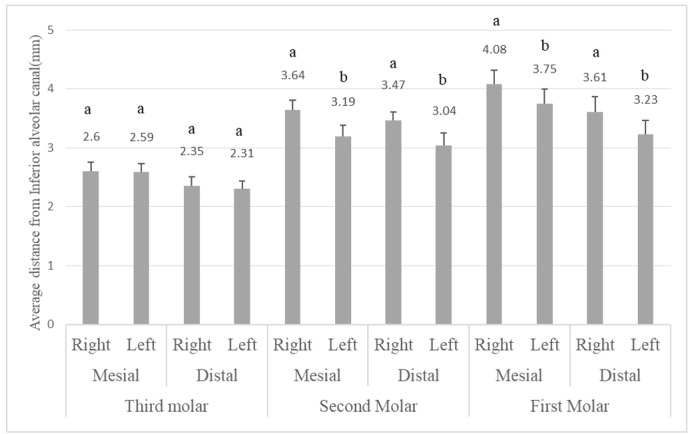
Figure 1. Average distances (mm) from mandibular molars to the inferior alveolar canal on the right and left sides, measured mesially and distally. Error bars represent standard deviations. Different letters (a, b) indicate significant differences (P<.05) based on statistical analysis.

In this study, 220 CBCT images were analyzed. Of these, 56.8% (n=125) were from female patients, and 43.2% (n=95) were from male patients. The age distribution showed 28.2% (n=62) of patients were under 18 years, 39.5% (n=87) were aged 18-49 years, and 32.3% (n=71) were over 49 years (Table-1).

The distances from mandibular molars and premolars to the IAC were measured mesially and distally on both the right and left sides (as shown in Figure-1). For the third molar, no significant differences were found between mesial and distal distances on either side (P>.05). For the second molar, the distal distance was significantly smaller than the mesial distance on both sides (right: P<0.001; left: P<0.001). Similarly, for the first molar, the distal distance was significantly smaller than the mesial distance (right: P=0.02; left: P=0.009). For the second premolar, the distance to the IAC was significantly greater on the right side compared to the left (P<.001), but no significant difference was observed for the first premolar (P=0.07), as shown in Table-2. A one-way ANOVA revealed significant differences in distances from the IAC across the five teeth (P<0.001). Duncan’s post-hoc test indicated that, on the right side, the first and second premolars had significantly greater distances than the molars (P<0.05), with the third molar having the smallest distance (P<0.05). On the left side, the first premolar had the greatest distance, and the third molar had the smallest (P<0.05). Gender comparisons showed no significant differences in third molar distances to the IAC for either side or measurement (P>0.05). However, for the second and first molars, distances were significantly greater in males than in females for both mesial and distal measurements on both sides (P<0.05). Similarly, for both premolars, distances were significantly greater in males than in females on both sides (P<0.001). Age group comparisons using one-way ANOVA showed no significant differences in distances to the IAC for any molars or premolars across the age groups (<18, 18-49, >49 years) on either side (P>.05). Table-3 presents the average distances from mandibular molars and premolars to the inferior alveolar canal (IAC) across age groups (49 years) for right and left sides, based on 220 CBCT images. Mesial and distal measurements are provided for molars, while premolars have a single measurement, with no significant differences (P>.05) observed across age groups per one-way ANOVA. Distances range from approximately 2.3-2.75 mm for third molars to 5.10-5.41 mm for first premolars, with standard deviations reflecting variability, indicating age does not notably affect tooth-IAC proximity (Table-4).

## Discussion

The location of the inferior alveolar canal, which houses the inferior alveolar nerve and the blood vessels that supply it, is vitally important to know for numerous dental operations. Avoiding inadvertent nerve damage requires precise understanding of the nerve's location within the bone and its connections to other anatomical features. Paresthesia, dysesthesia, and anesthesia of the inferior alveolar nerve are among the documented sensory abnormalities caused by these injuries [6]. Premolars on the right side (first and second) and the left side (first premolar) were discovered to be the furthest from the inferior alveolar canal, according to the current study's results. In the third molars, the distance from the inferior alveolar canal was the shortest on both the right and left sides.

The distance from the inferior alveolar canal to the mandibular third molars was measured by Rytkönen et al. (2018). Third molar extraction poses a risk of nerve injury to most lower teeth because of their proximity to the inferior alveolar canal [18]. This study's conclusions were corroborated by the present study. For both sexes, Adigüzel et al. (2012) found that distal roots were more closely spaced from the nerve than mesial roots [14]. There was a similar trend between the mesial and distal distances in this investigation.

According to this research, the distance between the right and left sides of the second premolar was larger than that between the first and first premolars, which were found to be of same size. Third molars on the right and left sides, as well as the distal and mesial features, did not differ significantly between men and women in this study. Nevertheless, the distance was noticeably larger in men compared to women in the first and second molars, on both the right and left sides, and in the mesial and distal aspects. Similarly, on both the right and left sides of the mouth, men had a noticeably longer distance from the tooth to the canal in the first and second premolars compared to women. Men and women of all ages differ significantly in the distance between the canal and the first and second mandibular molars, according to Vidya et al. (2019) [11].

According to research conducted by Simonton et al. (2009), women exhibited narrower mandibles at the distal and mesial apices in terms of overall width and shorter vertical lengths from the nerve to those apices [19]. The gender difference in the distance between the first and second molars as well as the second premolar was demonstrated by Hiremath et al. (2016). The current study's findings were in agreement with those of all these previous investigations [12]. But contrary to the current study's results, Adigüzel et al. [14] found that, across all age groups, men and women had identical distances from the inferior alveolar nerve to the root apices.

Age groups for any of the three molars on the right or left side, or on the distal or mesial side, did not differ significantly in the current study. There was no correlation between gender or age and the location of the mental foramen, as shown by Tafakhori et al. (2016) [20]. In a similar way, the relative location of the canal was virtually same across gender and age groups in the research of Angel et al. (2011) [21]. In contrast to the current study, Hiremath et al. (2016) found the opposite to be true. Based on age groupings, their investigation found a substantial difference in the distance between the first and second molars [12].

Adigüzel et al. (2012) found that the distance between the apex and the nerve was reduced in the age groups of 16-25 and 56-65, suggesting that the inferior alveolar nerve's distance from the root apices of the first mandibular molar varied with age [14]. Findings from the study by Simonton et al. (2009) about the relationship between the mandibular canal and the roots of the first mandibular molar were found to be predictive of gender and age [19].

The use of various radiographic image types may account for some of the discrepancies in the study outcomes. The tight link between the impacted mandibular third molar roots and the mandibular canal cannot be reliably predicted based on the presence or absence of radiographic signals in panoramic radiographs, as shown by Ishak et al. (2014) [22]. When it comes to accurately measuring the lengths from the apices of posterior teeth to the mandibular canal, Kim et al. (2010) found that CBCT is just as effective as anatomical slicing [23].

Because buccal bone is often removed by surgeons, it is possible that the canal may be injured during bone removal if it is located high vertically and appears to intersect with the tooth in panoramic pictures. Surgeons can determine the location of the nerve and plot a course for bone removal with the use of CBCT imaging [24]. According to research by Dabbaghi et al. (2014), which used cross-sectional cone beam computed tomography scans to study the inferior alveolar canal's position, this position remains largely stable regardless of gender or age [13].

The small number of instances studied could be a contributing factor to the conflicting study outcomes. Furthermore, other studies may have produced less reliable results due to imprecise definitions of the landmarks under investigation and the neglect to consider the impact of confounding variables on the measured variables. Discrepancies in study outcomes could be caused by using slices with different slice intervals in CBCT. Future research should use bigger samples, shorter age intervals, and a variety of racial and cultural groups to investigate additional mandibular canal-related landmarks.

## Conclusion

This study, conducted on an Iranian population, revealed distinct anatomical relationships between mandibular teeth and the IAC. The first and second premolars exhibited the greatest distances to the IAC on the right side, while the first premolar had the longest distance on the left. Conversely, the third molar consistently showed the shortest distance to the IAC bilaterally. No significant differences were observed between mesial and distal distances for third molars, but first and second molars displayed significantly shorter distal distances. Second premolars showed greater right-side distances compared to the left, unlike first premolars, which had similar distances bilaterally. Gender analysis indicated no differences for third molars, but males exhibited significantly greater distances for first and second molars and premolars compared to females. Age had no notable impact on these measurements, suggesting stable anatomical patterns across age groups in this Iranian cohort, providing baseline information for gauiding dental interventions in this population.

## Conflict of Interest

None.
